# From Ritual to Screen: Documenting *Tabuik* as Intangible Cultural Heritage

**DOI:** 10.12688/f1000research.184038.1

**Published:** 2026-07-06

**Authors:** Ananda Putriani, Sumiyadi Sumiyadi, Tedi Permadi, Halimah Halimah, Vereki Martiano, Nurmalinda Zari, Jamilah Aljah Siompu

**Affiliations:** 1 Universitas Pendidikan Indonesia, Bandung, West Java, Indonesia; 2 Universitas Putra Batam, Batam, Riau Islands, Indonesia; 3 Universitas Negeri Semarang, Semarang, Central Java, Indonesia

**Keywords:** Tabuik; intangible cultural heritage; cultural documentation; cultural representation; visual narrative.

## Abstract

Intangible cultural heritage (ICH) plays a crucial role in sustaining cultural identity, collective memory, and social continuity; however, existing studies have predominantly focused on preservation efforts and heritage policies, with limited attention to how documentation itself contributes to the construction of cultural meaning. This study examines the documentation of the *Tabuik* tradition in Pariaman, West Sumatra, as a process of cultural representation and meaning-making. Employing a qualitative autoethnographic approach, data were collected through participant observation, interactions with cultural practitioners, focus group discussions, and the development of a documentary narrative entitled *Tabuik Manusia: Dari Asal Menuju Melepaskan.* The data were analyzed using thematic analysis to identify patterns of representation and narrative construction within the documentation process. The findings reveal that documenting *Tabuik* extends beyond the technical recording of ritual activities and functions as an interpretive practice through which cultural meanings are selected, negotiated, and reconstructed. Four major themes emerged from the analysis: origin and loss as an existential foundation, silenced grief in adolescent experience, intergenerational expectations and identity negotiation, and responsibility as an embodied practice in ritual and everyday life. These themes demonstrate how ritual symbolism is translated into visual and narrative forms that connect collective cultural memory with contemporary personal experiences. The study argues that cultural documentation should be understood not merely as a preservation mechanism but as an epistemological and representational practice that actively shapes cultural understanding. By transforming ritual experiences into mediated narratives, documentation contributes to both the safeguarding of intangible cultural heritage and the ongoing production of cultural meaning, thereby highlighting the significance of reflexive and context-sensitive documentation in ensuring the relevance and sustainability of cultural heritage in contemporary society.

## Introduction

Intangible cultural heritage (ICH) plays a crucial role in sustaining collective identity, cultural memory, and social continuity across societies worldwide ( Harrison, 2013; Kirshenblatt-Gimblett, 2004; Kuutma, 2009; Smith, 2006; UNESCO, 2003). It encompasses practices, rituals, and cultural expressions transmitted across generations, serving as key markers of cultural diversity and social cohesion ( UNESCO, 2003). Within contemporary heritage studies, ICH is no longer understood as static inheritance, but rather as a dynamic social process that is continuously reconstructed in response to changing socio-cultural contexts ( Akagawa & Smith, 2019; Harrison, 2013). Recent studies also highlight that research on intangible cultural heritage has expanded significantly across disciplines, reflecting its growing importance in contemporary cultural discourse ( Hu et al., 2024; Pérez-Gandarillas, 2024).

However, amid accelerating processes of globalization and modernization, many traditional cultural practices face significant threats to their sustainability ( Logan, 2012; Silverman & Ruggles, 2007). These challenges not only risk the disappearance of cultural practices but also undermine the systems of meaning and identity embedded within them. Therefore, safeguarding intangible cultural heritage has become a central concern in contemporary cultural and heritage studies ( Kurin, 2004).

Rituals, as a fundamental component of intangible cultural heritage, function not only as symbolic practices but also as mechanisms for transmitting values, knowledge, and cultural identity across generations ( Bell, 2009; Turner, 1969). In recent years, scholarly attention has increasingly focused on the role of rituals within cultural heritage contexts, particularly in relation to community participation, cultural experience, and sustainability ( Hu et al., 2024). At the same time, rapid technological advancement has positioned cultural documentation as an essential strategy for ensuring the continuity of these practices. Documentation is no longer understood as a passive process of recording, but rather as an active and interpretive practice involving the selection, interpretation, and representation of cultural values ( Giaccardi, 2012). Empirical studies indicate that digital technologies and visual documentation significantly contribute to the preservation and dissemination of intangible cultural heritage, while also transforming how cultural practices are accessed and understood ( Dou et al., 2018; Ioannides et al., 2021; Zabulis et al., 2021).

Despite the growing attention to safeguarding intangible cultural heritage, existing studies suggest that documentation and preservation efforts continue to face conceptual limitations. Much of the research remains focused on identifying and describing cultural practices, without critically examining how documentation processes shape cultural meaning itself ( Akagawa & Smith, 2019; Kurin, 2004). Furthermore, cultural documentation is often treated as a neutral technical activity, whereas in practice it involves processes of selection, interpretation, and representation that influence how culture is understood and communicated ( Giaccardi, 2012). From a cultural studies perspective, representation is not merely a reflection of reality but a process through which meaning is constructed through language, symbols, and media ( Hall, 1997). Therefore, this study adopts a cultural representation perspective, which views documentation as a meaning-making process rather than a neutral recording activity.

In addition, recent developments in digital technology have introduced new challenges regarding how cultural practices can be represented without losing their social context and symbolic meanings ( Ioannides et al., 2021; Pandey & Kumar, 2020). While digital documentation enables wider access and preservation, it also raises critical questions about authenticity, interpretation, and the transformation of cultural practices in mediated forms. While previous studies have extensively examined preservation and policy aspects of intangible cultural heritage, limited attention has been given to how documentation practices actively construct cultural meaning through narrative and visual representation. This gap is particularly evident in studies focusing on local ritual traditions within contemporary socio-cultural transformations. Consequently, a significant research gap remains in examining cultural documentation not merely as a preservation tool, but as a process that constructs cultural representation and meaning within contemporary contexts.

In the Indonesian context, one form of intangible cultural heritage that continues to be practiced is the *Tabuik* tradition in Pariaman, West Sumatra. This annual ritual, performed during the month of Muharram, commemorates the historical events of Karbala and has evolved into a local cultural practice ( Asril, 2018; Dalmenda & Elian, 2017). Beyond its religious dimension, *Tabuik* functions as a medium for preserving cultural identity and collective memory within the community, reflecting the interrelationship between adat (customary practices) and religion in Minangkabau society ( Dalmenda & Elian, 2017). From a heritage perspective, such rituals serve not only as inherited traditions but also as dynamic practices that are continuously reinterpreted within changing social contexts ( Harrison, 2013; Smith, 2006). Based on this background, this study aims to analyze how the *Tabuik* tradition is documented as intangible cultural heritage within a contemporary societal context. Specifically, it examines documentation as a practice that involves processes of selection, interpretation, and narrative construction. This study seeks to explore how elements of the *Tabuik* ritual are translated into visual and narrative forms, and how these processes contribute to the preservation and understanding of cultural meaning.

This study offers a conceptual contribution by positioning cultural documentation as an interpretive and epistemological practice, rather than merely a technical activity of preservation. By integrating perspectives from heritage studies, ritual theory, and visual culture, this research highlights that documentation actively shapes cultural meaning, narratives, and representation. Furthermore, this study provides an empirical contribution through an analysis of the *Tabuik* tradition as a dynamic local cultural practice, thereby extending existing scholarship on the relationship between ritual, documentation, and cultural representation in contemporary society.

## Literature review

### Intangible cultural heritage (ICH)

Intangible cultural heritage is a key concept in contemporary cultural studies, referring to practices, representations, expressions, knowledge, and skills that are recognized by communities as part of their cultural heritage ( UNESCO, 2003). Unlike tangible cultural heritage, intangible cultural heritage is dynamic in nature and is continuously recreated by communities in response to their environment, social interactions, and historical experiences. Therefore, it should not be understood merely as a set of traditions passively inherited, but rather as living cultural practices that continuously evolve within changing social contexts ( Blake, 2015; Kurin, 2004). Recent studies also emphasize that intangible cultural heritage should be understood as a living and adaptive system that is continuously negotiated within contemporary societies ( Bortolotto, 2010; Hu et al., 2024; Pérez-Gandarillas, 2024; Su et al., 2019).

In the development of academic scholarship, intangible cultural heritage is no longer viewed solely as an object of preservation, but as a social construction that involves processes of interpretation, negotiation, and representation ( Bortolotto, 2010; Harrison, 2013; Smith, 2006). This perspective highlights that what is defined as “heritage” is not neutral; rather, it is shaped through power relations, cultural policies, and specific social and political interests. In this sense, heritage is actively produced through discourse and practice, rather than simply inherited from the past ( Smith, 2006). Consequently, safeguarding intangible cultural heritage is not only concerned with maintaining the continuity of cultural practices, but also with how cultural meanings are produced, mediated, and represented across different contexts ( Akagawa & Smith, 2019). Recent empirical research further shows that heritage practices are increasingly influenced by global cultural dynamics, digital transformation, and interdisciplinary approaches ( Chen et al., 2022; Hu et al., 2024).

Furthermore, in the context of globalization and modernization, intangible cultural heritage faces a range of challenges, including shifting social values, the commodification of culture, and changes in the functions of rituals within society ( Akagawa & Smith, 2019; Kurin, 2004; Logan, 2012). Contemporary studies also indicate that digital transformation and globalization processes significantly affect how cultural heritage is preserved and transmitted, often leading to reinterpretation and hybridization of cultural practices ( Ioannides et al., 2021; Qiu et al., 2022; Zabulis et al., 2021). On the one hand, preservation efforts are increasingly important for ensuring cultural sustainability; on the other hand, these processes may also transform the original meanings and functions of cultural practices. This tension reflects the complex relationship between preservation and transformation in contemporary heritage practices ( Blake, 2015). Therefore, the study of intangible cultural heritage cannot be separated from an analysis of dynamics of change, adaptation, and processes of cultural representation in contemporary societies.

### Cultural documentation

Cultural documentation constitutes a crucial aspect of efforts to safeguard intangible cultural heritage, particularly in the face of social change and technological development. In contemporary scholarship, documentation is no longer understood as a passive process of recording; rather, it is regarded as an active practice that plays a significant role in organizing, storing, and transmitting cultural knowledge to future generations ( Cameron & Kenderdine, 2007; Khan et al., 2018; Kurin, 2004). This is especially important given that intangible cultural heritage is inherently non-material and heavily dependent on social practices and collective memory, making it vulnerable to transformation or even disappearance in the absence of systematic documentation ( Kurin, 2004). Recent studies further emphasize that documentation practices are central to sustaining cultural continuity, particularly in rapidly changing societies ( Hu et al., 2024; Su et al., 2019).

With the advancement of digital technologies, cultural documentation practices have undergone significant transformation through processes such as digitization, database development, and cultural information systems. Studies suggest that digital documentation enables broader storage and dissemination of cultural knowledge; however, it also introduces challenges related to representation, interpretation, and cultural context ( Dou et al., 2018; Ioannides et al., 2021). In addition, research indicates that digital technologies not only preserve cultural heritage but also reshape how it is accessed, experienced, and interpreted by wider audiences ( Bekele et al., 2021; Rahaman et al., 2019; Zabulis et al., 2021). In this context, documentation functions not only as a tool for preservation but also as a medium that mediates the relationship between culture, technology, and society.

Furthermore, a number of studies emphasize that cultural documentation is not neutral, as it inherently involves processes of selection, interpretation, and the construction of cultural representation ( Giaccardi, 2012)vv. Documentation, particularly in visual and digital forms, plays a significant role in shaping how cultural practices are understood and represented within broader contexts. From a contemporary perspective, digital documentation also raises critical questions regarding authenticity, ownership, and the transformation of cultural meaning in mediated environments ( Jones, 2017; Pandey & Kumar, 2020; Zabulis et al., 2021). Therefore, the documentation of intangible cultural heritage is not solely concerned with preservation, but also with the production of meaning and cultural representation in contemporary society.

### Representation and visual culture

Cultural representation constitutes a significant aspect in the study of cultural heritage, particularly in understanding how cultural practices are produced, interpreted, and communicated across various social contexts. From a cultural studies perspective, representation is not understood as a neutral reflection of reality; rather, it is a process of meaning construction through language, symbols, and media ( Hall, 1997). Therefore, any form of cultural documentation, especially those based on visual media, inherently involves processes of selection and interpretation that shape how a culture is understood by broader audiences. Recent studies further highlight that representation plays a crucial role in shaping public understanding of cultural heritage, particularly in mediated and digital contexts ( Smith & Waterton, 2009; Waterton & Smith, 2010; Waterton & Watson, 2015).

Within the context of visual culture, media such as documentary film, photography, and digital platforms play a crucial role in representing cultural practices. Visual representation functions not only as a tool of documentation but also as a medium that constructs cultural narratives and meanings through specific visual choices, narrative structures, and perspectives ( Pink, 2013)vv. Empirical research in visual and digital heritage studies suggests that media technologies significantly influence how cultural experiences are framed, accessed, and interpreted by audiences ( Champion, 2020; Ioannides et al., 2021; Rahaman et al., 2019). In addition, studies in visual anthropology and media research emphasize that images and visual narratives are not neutral representations but are embedded within cultural, political, and epistemological frameworks ( Banks, 2018; Banks & Ruby, 2011). Consequently, visual documentation cannot be separated from interpretation, as every cultural representation is inevitably influenced by the perspective of its creator.

Furthermore, in contemporary studies, cultural representation through visual media is closely linked to issues of authenticity, power, and the construction of cultural identity. Several studies indicate that representation processes can reinforce, transform, or even simplify particular cultural meanings, depending on how such cultures are selected and presented ( Rose, 2016). In addition, recent scholarship highlights that digital and visual representations may reshape cultural meanings through processes of mediation, recontextualization, and audience interaction ( Bekele et al., 2021; Jones, 2017; Zabulis et al., 2021). In the context of intangible cultural heritage, this becomes especially significant, as visual documentation not only records cultural practices but also contributes to shaping collective understandings of those practices. Thus, the study of cultural documentation cannot be detached from an analysis of visual representation as a process of meaning-making within contemporary society.

## Methodology

This study employs a qualitative approach with an autoethnographic perspective to analyze the documentation of the *Tabuik* tradition as a practice of representing intangible cultural heritage. A qualitative approach is adopted as it allows for an in-depth exploration of meanings, experiences, and social processes within specific cultural contexts ( Creswell, 2014). Meanwhile, autoethnography is utilized as a reflective approach that connects the researcher’s personal experience with broader cultural analysis, enabling a more contextualized understanding of cultural representation practices ( Ellis et al., 2011). The study is conducted in Pariaman City, West Sumatra, which serves as the central location for the *Tabuik* tradition. Data are collected through direct observation of the *Tabuik* ritual sequence, particularly the Maambiak Tanah and Buang Laut processions. In addition, data are obtained through interactions with cultural actors, including traditional elders (Tuo *Tabuik*) and youth who actively participate in the social practices surrounding the ritual. One of the primary data sources in this study is the development of a documentary storyline entitled *Tabuik* Manusia: Dari Asal Menuju Melepaskan, which is constructed based on field observations, focus group discussions (FGDs), and the researcher’s reflective experiences throughout the documentation process.

Prior to participation, all participants provided verbal informed consent. Verbal consent was considered more appropriate than written consent because the study was conducted in a community-based cultural setting where interactions were informal and rooted in local customary practices. Some participants, particularly traditional elders, were more comfortable providing oral agreement rather than signing formal documents. Before data collection, participants were informed about the objectives of the study, the use of visual documentation, the voluntary nature of their participation, and their right to withdraw at any stage without consequence. To protect participant privacy, all names were replaced with pseudonyms and identifying information was removed or anonymized during data analysis and publication.

This storyline is positioned as qualitative data that represents processes of selection, interpretation, and meaning construction within cultural documentation practices. Within the autoethnographic framework, the researcher acts as both the primary instrument and an active participant directly involved in the documentation process. The researcher’s position is inherently reflective, acknowledging that personal experiences, background, and engagement shape the interpretation of the data. This approach emphasizes the importance of reflexivity in qualitative research, defined as a critical awareness of the researcher’s positionality in the production of knowledge ( Ellis et al., 2011) Ellis. To enhance data credibility, this study employs source triangulation by comparing observational data, field notes, and insights obtained from discussions with cultural practitioners. Triangulation is used to ensure interpretive consistency and to strengthen the validity of the findings in qualitative research ( Creswell, 2014). Data analysis is conducted using thematic analysis by identifying patterns of cultural representation within the documentation process of the *Tabuik* ritual. The analytical process includes coding, theme development, and interpretation of meanings. This approach enables the researcher to uncover underlying structures of meaning within the data and to understand how ritual elements are translated into visual and narrative forms ( Braun & Clarke, 2006). Thus, the analysis extends beyond mere data description to examine the construction of cultural representation within documentation practices.

## Findings

### Origin and loss as an existential foundation

The findings of this study indicate that the *Tabuik* ritual functions not merely as a cultural practice but also as an existential framework for understanding the origins of life, loss, and the continuity of human meaning. One of the key elements of this ritual is the Maambiak Tanah procession, which symbolically represents the origin of human life from the earth.

This symbolic meaning becomes particularly significant when connected to the personal experience of the character Participant 1, who undergoes the loss of a father figure as the foundation of his existence. The interrelation between ritual symbolism and personal experience suggests that loss is not solely an individual emotional condition but is mediated through cultural symbolic systems. This is reflected in Participant 1’s statement that he felt “lost since his father’s passing,” indicating that the experience of loss is not only emotional but also existential in nature. In this context, the earth as a symbol of origin does not merely signify the beginning of life but also embodies notions of separation and human limitation.

Furthermore, these findings demonstrate that the experience of loss within personal narratives can be understood as part of a broader structure of meaning embedded in cultural practices. The *Tabuik* ritual provides a symbolic framework that enables individuals like Participant 1 to interpret loss as part of the cycle of life, rather than as a fragmented or isolated experience. Thus, personal experience does not stand alone but is interconnected with collective narratives transmitted through cultural traditions.

Within the process of documentation, these symbolic meanings undergo transformation through the construction of visual narratives. The Maambiak Tanah procession is not only documented as a ritual event but is also represented as a metaphor for Participant 1’s experience of loss. This narrative choice demonstrates that documentation is not neutral; rather, it actively shapes the relationship between collective memory and individual experience.

Thus, the relationship between origin and loss emerges as a thematic foundation that reveals the intersection of ritual, personal experience, and visual representation. These findings affirm that cultural documentation does not merely record cultural practices but also contributes to constructing an existential understanding of life, loss, and meaning within contemporary society.

### Silenced grief in adolescent experience

The findings of this study indicate that grief experienced by adolescents in this context tends not to be expressed openly, but rather internalized and lived through in private spaces. Grief is not manifested through explicit emotional expressions in public settings; instead, it appears in the form of silence, withdrawal, and deep internal reflection.

This is evident in Participant 2’s statement that he “has never truly been able to accept the loss,” suggesting that experiences of loss are not always verbally articulated but are embodied through implicit and internal emotional processes. In this context, the grief experienced by Participant 2 and other adolescents is not solely individual but is also shaped by social and cultural norms that regulate how emotions are expressed. The tendency to conceal grief can be understood as part of the internalization of social values that position certain emotional expressions as something to be controlled. Consequently, grief does not emerge as a shared collective experience but rather as a personal experience that is endured silently.

Furthermore, the findings indicate that private spaces such as bedrooms, beaches, and other personal environments become significant sites for the expression of grief. Within these spaces, individuals like Participant 2 have the freedom to reflect on their experiences of loss without social pressure. This suggests that grief does not disappear but instead transforms into a more reflective and introspective experience.

In the process of documentation, this silenced grief is not represented in a dramatic manner but through subtle visual approaches and contemplative narrative structures. The decision to avoid explicit emotional displays becomes a representational strategy that reinforces the understanding of grief as a complex experience that cannot always be directly expressed. Thus, documentation does not merely capture grief but actively shapes how it is interpreted by the audience.

These findings affirm that grief in adolescence is not merely an emotional response to loss but constitutes a social and cultural experience shaped by norms, spatial contexts, and representational processes. Therefore, silenced grief can be understood as an alternative form of expression that reflects the interplay between individual experience, social structures, and practices of cultural documentation.

### Intergenerational expectations and identity negotiation

The findings of this study indicate that adolescent life in this context is closely shaped by intergenerational expectations, particularly those originating from parents and the surrounding social environment. These expectations are associated with educational choices, career paths, and life trajectories considered ideal. In many cases, adolescents are confronted with pressures to follow predetermined paths that do not necessarily align with their personal interests or aspirations. This is reflected in the experience of the character Queen, who states that she feels “obliged to follow choices that have already been determined, even though they are not what I want,” revealing a tension between individual agency and familial expectations.

In this context, adolescent identity is not formed autonomously but emerges through a process of negotiation between personal desires and the social structures that shape them. The pressure to fulfill family expectations can be understood as part of a social mechanism through which values, norms, and aspirations are transmitted across generations. This conflict is also evident in other characters who experience uncertainty in making life decisions, positioned between personal aspirations and strong familial demands.

These findings become increasingly significant when connected to the cultural practice of *Tabuik*, which also involves the intergenerational transmission of values and roles. The involvement of traditional leaders (Tuo *Tabuik*) in preserving and continuing the ritual reflects how culture is sustained through robust social structures. In this regard, a parallel can be drawn between the transmission of cultural values within the *Tabuik* ritual and the transmission of expectations in adolescent life, both of which require the continuity of values across generations.

In the process of documentation, the tension between expectations and identity is represented through narratives that implicitly depict the characters’ internal conflicts. Rather than presenting explicit resistance, the representation emphasizes reflection and meaning-making processes undertaken by the characters. This demonstrates that documentation is not merely a neutral recording of social reality but actively constructs narratives about how individuals negotiate their identities within the context of social and cultural pressures.

Thus, these findings affirm that intergenerational expectations constitute a significant factor in shaping adolescent experiences and identities. The process of identity negotiation occurs not only at the individual level but is also embedded within broader social structures and cultural practices. Therefore, cultural documentation functions as a medium that not only represents these tensions but also reveals the dynamic relationship between individuals, families, and culture within contemporary society.

### Responsibility as an embodied practice in ritual and everyday life

The findings of this study indicate that responsibility, within this context, is not merely understood as a normative concept but as a practice enacted through the body and everyday activities. In the *Tabuik* tradition, responsibility is manifested through the physical engagement of community members, including the construction of the *Tabuik*, collective labor, and participation in ritual processes. In this sense, responsibility functions not only as a social obligation but also as a lived cultural practice transmitted across generations.

This dimension of responsibility as an embodied practice is also reflected in adolescent life, particularly in the experience of Participant 3, who actively contributes to the family economy through trading activities.

Participant 3 expresses that he “must continue to carry out his responsibilities, even when circumstances are not always easy,” indicating that responsibility is not merely a matter of choice but an integral part of self-formation and maturation. In this context, responsibility emerges as a concrete experience enacted through action rather than as an abstract value. The relationship between ritual practice and everyday life reveals a parallel between responsibility in cultural contexts and responsibility in personal life. While in the *Tabuik* ritual responsibility is realized through collective labor and devotion to tradition, in adolescent life it takes the form of effort, work, and personal sacrifice. Thus, responsibility can be understood as a practice that connects individuals to their communities while bridging the present with cultural heritage.

In the process of documentation, this dimension of responsibility is represented through visualizations that emphasize movement, labor, and bodily engagement. Such representations do not merely present final outcomes but also foreground the processes and dynamics involved. This demonstrates that documentation plays an active role in presenting responsibility as an embodied practice one that is physically experienced and enacted rather than merely symbolically represented.

These findings affirm that responsibility operates as a practice that connects ritual, everyday life, and processes of cultural representation. Responsibility is not only transmitted as a value but is enacted through concrete actions that shape both individual and collective experiences. Therefore, cultural documentation does not merely record these practices but also reveals how responsibility is lived as part of cultural continuity in contemporary society.

## Discussion

This study argues that the documentation of intangible cultural heritage should not be understood as a neutral act of representation, but as a cultural practice that actively constructs meaning through processes of selection, interpretation, and visual narration. This perspective aligns with previous scholarship that positions heritage as something continuously produced through social practices and representational frameworks ( Harrison, 2013; Smith, 2006). In the context of the *Tabuik* tradition, documentation does not merely record ritual sequences as cultural events, but operates as a site of meaning-making that connects ritual symbolism with subjective experiences such as loss, identity, and responsibility. In this sense, documentation functions as a mediating practice that bridges collective memory and personal experience in contemporary society ( Hall, 1997).

Furthermore, the findings support the conceptualization of intangible cultural heritage as a dynamic and continuously recreated process, as emphasized in the UNESCO Convention ( UNESCO, 2003). Rather than serving solely as a preservation tool, documentation becomes part of the ongoing reconstruction of cultural meaning. This aligns with the view that heritage depends on evolving social practices and interpretations ( Kurin, 2004). Thus, the documentation of *Tabuik* can be understood as an act of cultural actualization, where ritual meanings are not only maintained but also rearticulated through visual and narrative forms.

From a representation perspective, meaning is not inherent but produced through representational practices ( Hall, 1997). The documentation of *Tabuik* therefore cannot be treated as an objective reflection of cultural reality, but as an interpretive process shaped by narrative decisions, visual framing, and contextual positioning. The selection of scenes and the construction of narrative structures demonstrate that documentation actively shapes how culture is understood rather than simply presenting it.

Importantly, the findings also reveal that cultural representation is not static but open to continuous reinterpretation. The *Tabuik* ritual, traditionally rooted in religious and historical meanings, undergoes a shift when represented through visual narratives that connect it to contemporary youth experiences. This indicates that documentation does not merely reproduce existing meanings but generates new interpretive possibilities relevant to present social contexts.

The themes of silenced grief, identity negotiation, and responsibility as embodied practice further demonstrate that individual experiences are inseparable from broader social and cultural structures. Experiences such as loss, familial expectations, and responsibility are shaped through complex social relations ( Giddens, 1991; Hall, 1996). In this study, grief is internalized rather than publicly expressed, reflecting social norms governing emotional expression, while intergenerational expectations reveal identity as a negotiated process rather than an autonomous formation. Meanwhile, responsibility emerges not only as a symbolic value but as an embodied practice enacted in everyday life.

This study contributes conceptually by repositioning cultural documentation as an epistemological practice rather than a passive archival process. Unlike conventional approaches that treat documentation as a recording tool, this research demonstrates that documentation actively constructs relationships between ritual, personal experience, and social meaning through visual narratives. It also extends intangible cultural heritage studies by integrating autoethnographic and visual documentation perspectives, offering a more reflexive understanding of how subjectivity shapes cultural representation.

Finally, these findings have important implications for heritage studies and documentary practices. An interpretive approach to documentation enables cultural heritage to remain dynamic and meaningful across generations, rather than fixed and static ( UNESCO, 2003). Moreover, documentation can be understood as a dialogical space that connects tradition and modernity, collective memory and individual experience. This suggests that reflexive and context-sensitive documentation practices are essential for sustaining intangible cultural heritage in increasingly complex contemporary societies.

## Conclusion

This study argues that the documentation of intangible cultural heritage cannot be understood as a neutral process, but rather as a cultural practice that actively constructs meaning through visual representation and narrative. In the context of the *Tabuik* tradition, documentation functions as a mediating space that connects ritual symbolism with personal experiences, including loss, identity, and responsibility in adolescent life. These findings demonstrate that cultural meaning is not statically inherited but continuously negotiated and reconstructed through documentation practices in contemporary society. The main contribution of this research lies in positioning cultural documentation as both an interpretative and epistemological practice that shapes how culture is understood and represented. By integrating autoethnographic perspectives with visual documentation, this study not only expands the field of intangible cultural heritage studies but also offers a more reflexive approach to understanding the relationship between ritual, individual experience, and the production of cultural meaning. More broadly, this study suggests that a reflective and context-sensitive approach to documentation can serve as a crucial strategy for sustaining intangible cultural heritage amid ongoing social change. Therefore, cultural documentation should not be viewed merely as a tool for preservation, but as a cultural practice that enables heritage to remain dynamic, relevant, and meaningful for present and future generations.

## AI Declaration statetment

During the preparation of this manuscript, AI-assisted tools were used solely for language improvement, grammar checking, literature exploration, and reference support. All processes of data interpretation, analysis, argument development, and the originality of the work were conducted independently by the author. The author fully reviewed and verified all content and takes complete responsibility for the final version of the manuscript.

## Data Availability

This study employed a qualitative research design involving customary leaders, cultural stakeholders, and community representatives as research participants. The data consist primarily of interview transcripts, field notes, and documentation related to cultural knowledge and practices associated with the *Tabuik* tradition. The underlying raw data are not publicly available due to ethical, privacy, and cultural considerations. Participants did not provide consent for the public release of unprocessed interview materials, and several informants explicitly requested that culturally sensitive information shared during the research process remain restricted. Public disclosure of the raw data could compromise participant confidentiality and the integrity of culturally significant knowledge entrusted to the researchers. Furthermore, this study does not generate a quantitative dataset that can be openly shared or reused in the conventional sense. The findings are based on qualitative interpretations of participant narratives and cultural documentation. All relevant information necessary to understand and evaluate the study’s conclusions is included within the article. Researchers who wish to access additional data or supporting materials may submit a reasonable request to the corresponding author, Ananda Putriani, via email at ananda.putriani@upi.edu. Access may be granted subject to ethical approval, participant consent, and considerations regarding the protection of confidential and culturally sensitive information.
