# Supplementary material for: From Ritual to Screen: Documenting Tabuik as Intangible Cultural Heritage
Source: F1000Res. 2026 Sep 1;15:1082. Originally published 2026 Jul 6. [Version 2] doi: 10.12688/f1000research.184038.2 (PMC13583351; doi:10.12688/f1000research.184038.2)
Supplement: Peer review history

# Reviewer report: Reviewer response for version 2

**DOI:** 10.5256/f1000research.208883.r516964

**Published:** 2026-09-15

**Authors:** Munyika Sibanda

**Affiliations:** 1 Great Zimbabwe University, Masvingo, Zimbabwe

No further comments. APPROVED

Is the work clearly and accurately presented and does it cite the current literature?

Partly

If applicable, is the statistical analysis and its interpretation appropriate?

Not applicable

Are all the source data underlying the results available to ensure full reproducibility?

No source data required

Is the study design appropriate and is the work technically sound?

Partly

Are the conclusions drawn adequately supported by the results?

Partly

Are sufficient details of methods and analysis provided to allow replication by others?

No

Reviewer Expertise:

Heritage management, Museology, Records and Archives Management, Archaeology

I confirm that I have read this submission and believe that I have an appropriate level of expertise to confirm that it is of an acceptable scientific standard.

# Reviewer report: Reviewer response for version 1

**DOI:** 10.5256/f1000research.203145.r500663

**Published:** 2026-08-07

**Authors:** Munyika Sibanda

**Affiliations:** 1 Great Zimbabwe University, Masvingo, Zimbabwe

**Summary:** This autoethnographic study examines documentation of the Tabuik ritual (Pariaman, West Sumatra) as an interpretive, meaning-making practice rather than neutral recording, built around a documentary project ("Tabuik Manusia"). Thematic analysis yields four themes which are; origin/loss, silenced adolescent grief, intergenerational identity negotiation, and responsibility as embodied practice, each linking ritual symbolism to an individual's personal narrative. The theoretical framing (Hall's representation theory, Smith/Harrison on heritage as social construction) is well-integrated and the paper makes a genuine conceptual contribution to ICH documentation studies.

**Points that must be addressed:**

1. **Clarify participant status and data provenance.** State explicitly whether "Participant 1/2/3" and "Queen" are real research informants and clarify why naming is inconsistent (numbered vs. named). Specify whether quoted statements are verbatim interview data or dramatized/scripted documentary lines. This is the single most important fix as it determines whether the findings section is being presented as empirical or creative-interpretive work.
2. **Add methodological detail for replicability**: number of participants, selection criteria, fieldwork duration, number of FGDs and their composition.
3. **Correct the Rahaman et al. (2019) citation;** it does not support the claims it's attached to; replace with an appropriate heritage/digital-documentation source. It is a likely hallucinated/mis attached citation which requires correction before indexing can be considered sound.
4. **Broaden the evidentiary base per theme or** explicitly frame the findings as illustrative case vignettes rather than generalized patterns, to better match the single-quote-per-theme structure.
5. **Clean up editing artifacts**: "(Giaccardi, 2012)vv" (×2) and "(Ellis et al., 2011) Ellis."
6. **Add a Limitations section** addressing the small/unclear sample and the researcher's dual role as both documentarian and analyst.

Is the work clearly and accurately presented and does it cite the current literature?

Partly

If applicable, is the statistical analysis and its interpretation appropriate?

Not applicable

Are all the source data underlying the results available to ensure full reproducibility?

No source data required

Is the study design appropriate and is the work technically sound?

Partly

Are the conclusions drawn adequately supported by the results?

Partly

Are sufficient details of methods and analysis provided to allow replication by others?

No

Reviewer Expertise:

Heritage management, Museology, Records and Archives Management, Archaeology

I confirm that I have read this submission and believe that I have an appropriate level of expertise to confirm that it is of an acceptable scientific standard, however I have significant reservations, as outlined above.

# Response

**Published:** 2026-08-17

**Authors:** Ananda Putriani

**Affiliations:** Universitas Pendidikan Indonesia Sekolah Pascasarjana, Bandung, West Java, Indonesia

**Response to Reviewer**

 We sincerely thank the reviewer for the careful and constructive comments. We have thoroughly revised the manuscript in response to all of the points raised. The revisions have particularly clarified the participant status and data provenance, strengthened the methodological description, corrected the citation issue, reframed the findings as illustrative narrative cases rather than generalized patterns, removed the identified editing artifacts, and added a limitations section addressing the scope of the study and the researcher’s dual role. Our detailed responses are provided below.

**Comment 1. Clarify participant status and data provenance.**

***Reviewer comment:***

 State explicitly whether “Participant 1/2/3” and “Queen” are real research informants and clarify why naming is inconsistent (numbered vs. named). Specify whether quoted statements are verbatim interview data or dramatized/scripted documentary lines. This is the single most important fix as it determines whether the findings section is being presented as empirical or creative-interpretive work.

***Response:***

 Thank you for highlighting this important issue. We have clarified the status and provenance of the participants and the narrative data in the revised manuscript. The study involved 36 individuals as sources of research data, consisting of 30 senior high school students who participated in FGDs and six tradition bearers/practitioners. Nine of the 30 student participants were subsequently selected for thematic coding because their personal narratives were sufficiently detailed and relevant to the documentary themes. These nine students are a subset of the 30 FGD participants and are not an additional participant group.

 We have also clarified that “Participant 1,” “Participant 2,” and “Participant 3” refer to anonymized student participants whose personal narratives were used as illustrative cases in the findings. The use of numbered labels is intended to protect participant confidentiality and maintain consistency with the ethical procedures described in the methodology. We have further clarified that the findings are not intended to present these narratives as generalized representations of all adolescents or Tabuik participants.

 The quoted statements in the findings are derived from participants’ personal narratives and are presented as part of the qualitative interpretation of their experiences. At the same time, we have clarified the distinction between participants’ narrative accounts and the subsequent documentary construction. The documentary storyline represents an interpretive and creative transformation of the field data rather than a claim that every narrative element appearing in the documentary is a verbatim statement from participants.

 These clarifications have been incorporated into the Methodology and Findings sections. The revised methodology now explicitly describes the participant structure, selection process, anonymization, and the role of the documentary storyline in the analytical process.

**Comment 2. Add methodological detail for replicability.**

***Reviewer comment:***

 Provide the number of participants, selection criteria, fieldwork duration, number of FGDs, and their composition.

***Response:***

 We appreciate this suggestion and have substantially expanded the Methodology section. The revised manuscript now specifies that the study involved 36 participants, comprising 30 senior high school students participating in FGDs and six tradition bearers/practitioners. The six tradition bearers consisted of two Tuo Tabuik, one Maambiak Tanah diver, one elder woman involved in the tradition, one gandang tasa practitioner, and one ninik mamak.

 We have also clarified the selection criteria. Participants were purposively selected based on their direct involvement in, familiarity with, or cultural expertise regarding the Tabuik ritual. The revised manuscript further explains that nine students were selected from the 30 FGD participants for thematic coding because their personal narratives provided sufficiently detailed and relevant accounts for the development of the documentary themes.

 In addition, Tables 1–3 have been added/revised to provide greater transparency regarding participant composition, demographic characteristics, school affiliation, and the analytical structure of the participant group. We also clarify the approximate duration of participant interactions, which was 20–30 minutes, and distinguish the student narrative subset from the broader FGD group.

**Comment 3. Correct the Rahaman et al. (2019) citation.**

***Reviewer comment:***

 The citation does not support the claims to which it is attached. Replace it with an appropriate heritage/digital-documentation source.

***Response:***

 Thank you for identifying this citation problem. We have reviewed the relevant discussion and corrected the citation. The unsupported/mis attached citation has been removed from the relevant argument and replaced with references that more directly support the discussion of visual representation, digital heritage, and cultural documentation. The revised literature review now relies on more appropriate sources, including Ioannides et al. (2021), Bekele et al. (2021), Zabulis et al. (2021), and other relevant heritage and visual-culture scholarship.

 We have also carefully checked the references and in-text citations throughout the manuscript to reduce the possibility of citation misalignment.

**Comment 4. Broaden the evidentiary base per theme or explicitly frame the findings as illustrative case vignettes.**

***Reviewer comment:***

 The findings should either be supported by a broader evidentiary base or explicitly framed as illustrative case vignettes rather than generalized patterns.

***Response:***

 We agree with the reviewer’s concern and have revised the framing of the Findings section accordingly. Rather than presenting the four themes as generalized patterns applicable to all participants, we now explicitly identify them as illustrative narrative cases developed through thematic analysis of nine student narratives selected from the broader group of 30 FGD participants.

 A clarification has been added at the beginning of the Findings section stating that the four themes are interpreted as illustrative narrative cases rather than generalized patterns across all participants. Each vignette is therefore intended to foreground a particular relationship between an individual experience, the symbolic structure of the Tabuik ritual, and the documentary choices through which these elements were connected.

 We have also revised the language within individual findings to avoid overgeneralization. For example, the discussion of Participant 2 explicitly states that the account should not be interpreted as evidence that all adolescents' experience grief in the same way, but rather as a situated narrative demonstrating how personal loss may be negotiated through silence, withdrawal, and private reflection.

 This revised framing is consistent with the study’s qualitative autoethnographic and interpretive orientation and better reflects the evidentiary scope of the data.

**Comment 5. Clean up editing artifacts.**

***Reviewer comment:***

 Remove “(Giaccardi, 2012)vv” (×2) and “(Ellis et al., 2011) Ellis.”

***Response:***

 Thank you for pointing out these editorial errors. We have carefully proofread the manuscript and removed the identified editing artifacts, including the duplicated “vv” following the Giaccardi (2012) citation and the erroneous duplication associated with Ellis et al. (2011). The relevant citations and surrounding sentences have also been checked for consistency and grammatical accuracy.

**Comment 6. Add a Limitations section.**

***Reviewer comment:***

 Add a Limitations section addressing the small/unclear sample and the researcher’s dual role as both documentarian and analyst.

***Response:***

 We agree with the reviewer and have added a dedicated Limitations section to explicitly acknowledge the scope and methodological limitations of the study. The revised section clarifies that the study is interpretive and case-oriented, and therefore does not seek statistical or broad empirical generalization. It also acknowledges the researcher’s dual role as documentarian and analyst within the autoethnographic framework.

 In particular, the limitations section explains that the interpretation of participants’ narratives and the transformation of those narratives into documentary forms are inevitably influenced by the researcher’s positionality, selection of scenes, narrative framing, and representational decisions. We acknowledge that this dual role may introduce interpretive subjectivity, although reflexivity and source triangulation were employed to strengthen the credibility of the analysis.

 The revised Limitations section also clarifies that the nine student narratives used for thematic coding represent a selected subset of the broader participant group and should therefore be understood as illustrative rather than representative of all adolescents or Tabuik practitioners.

 We believe that these revisions provide a clearer alignment between the study’s methodological design, evidentiary scope, and interpretive claims.

 We sincerely appreciate the reviewer’s detailed comments, which have helped us improve the methodological transparency, evidentiary framing, and overall clarity of the manuscript. We believe that the revised manuscript now more clearly distinguishes empirical participant narratives from documentary interpretation and appropriately positions the findings as illustrative cases within an autoethnographic and interpretive framework. We hope that the revisions adequately address the reviewer’s concerns and improve the manuscript’s suitability for further consideration.

# Reviewer report: Reviewer response for version 1

**DOI:** 10.5256/f1000research.203145.r500665

**Published:** 2026-07-21

**Authors:** Libra Dui Putra

**Affiliations:** 1 Universitas Gadjah Mada, Yogyakarta, Indonesia

This manuscript is well structured, clearly written, and addresses an important issue in the field of intangible cultural heritage. The study makes a meaningful contribution by moving beyond the conventional perspective of cultural documentation as merely a preservation tool and instead conceptualizing documentation as an epistemological and representational practice that actively constructs cultural meaning. This perspective provides an original theoretical contribution to the literature on intangible cultural heritage, cultural representation, and memory studies.

 The integration of an autoethnographic approach with thematic analysis is appropriate for addressing the research objectives and offers rich insights into how the Tabuik tradition is interpreted, negotiated, and communicated through documentary media. The identification of the four thematic dimensions strengthens the analytical depth of the study and demonstrates the complex relationship between ritual, identity, collective memory, and contemporary cultural experience.

 Overall, this article has the potential to make a significant contribution to the development of theory and scholarship in cultural heritage studies, visual anthropology, cultural

 documentation, and related interdisciplinary fields. The manuscript is scientifically sound, methodologically appropriate, and suitable for publication. I therefore recommend

 Approved.

Is the work clearly and accurately presented and does it cite the current literature?

Yes

If applicable, is the statistical analysis and its interpretation appropriate?

Yes

Are all the source data underlying the results available to ensure full reproducibility?

No source data required

Is the study design appropriate and is the work technically sound?

Yes

Are the conclusions drawn adequately supported by the results?

Yes

Are sufficient details of methods and analysis provided to allow replication by others?

Yes

Reviewer Expertise:

culture, linguistics, gender studies, and toponymy. My review was conducted primarily from these disciplinary perspectives.

I confirm that I have read this submission and believe that I have an appropriate level of expertise to confirm that it is of an acceptable scientific standard.
